# Bioinformatics reveal elevated levels of Myosin Vb in uterine corpus endometrial carcinoma patients which correlates to increased cell metabolism and poor prognosis

**DOI:** 10.1371/journal.pone.0280428

**Published:** 2023-01-20

**Authors:** Kristen A. Engevik, Melinda A. Engevik, Amy C. Engevik

**Affiliations:** 1 Department of Molecular Virology & Microbiology, Baylor College of Medicine, Houston, Texas, United States of America; 2 Department of Regenerative Medicine & Cell Biology, Medical University of South Carolina, Charleston, South Carolina, United States of America; 3 Department of Microbiology & Immunology, Medical University of South Carolina, Charleston, South Carolina, United States of America; BMSCE: BMS College of Engineering, INDIA

## Abstract

Carcinoma of the endometrium of the uterus is the most common female pelvic malignancy. Although uterine corpus endometrial cancer (UCEC) has a favorable prognosis if removed early, patients with advanced tumor stages have a low survival rate. These facts highlight the importance of understanding UCEC biology. Computational analysis of RNA-sequencing data from UCEC patients revealed that the molecular motor Myosin Vb (MYO5B) was elevated in the beginning stages of UCEC and occurred in all patients regardless of tumor stage, tumor type, age, menopause status or ethnicity. Although several mutations were identified in the MYO5B gene in UCEC patients, these mutations did not correlate with mRNA expression. Examination of MYO5B methylation revealed that UCEC patients had undermethylated MYO5B and undermethylation was positively correlated with increased mRNA and protein levels. Immunostaining confirmed elevated levels of apical MYO5B in UCEC patients compared to adjacent tissue. UCEC patients with high expressing MYO5B tumors had far worse prognosis than UCEC patients with low expressing MYO5B tumors, as reflected by survival curves. Metabolic pathway analysis revealed significant alterations in metabolism pathways in UCE patients and key metabolism genes were positively correlated with MYO5B mRNA. These data provide the first evidence that MYO5B may participate in UCEC tumor development.

## Introduction

Uterine cancer is a common gynecologic cancer worldwide (Global Health Data Exchange (GHDx)). The incidence of uterine cancer cases increased 2.3 times from 1990 to 2019, and uterine cancer resulted in 91,640.7 deaths globally in 2019. There are two primary types of uterine cancer: endometrial cancer and uterine sarcoma. Endometrial cancer is the most common type of uterine cancer, making up 90% of cases, and can be further divided into subtypes based on tumor histology. Uterine corpus endometrial carcinoma (UCEC), a subset of endometrial cancer, is a common malignancy in women [1]. It is estimated that women have a 2.6% lifetime risk of developing UCEC [2] and this cancer commonly occurs in women 60 years or older [3]. Importantly, UCEC is currently the fourth most life-threatening cancer in females, after ovarian cancer and cervical cancer [4]. Fortunately, approximately 75% of UCEC patients are diagnosed early, resulting in a 5-year survival rate of more than 70% [5–7]. However, the prognosis of UCEC patients with grade III endometrioid cancer, serous carcinoma, and mixed adenocarcinoma is still poor with a 5-year survival rate of 17% [5, 8–10]. Additionally, the risk of metastasis and recurrence is also high in these patients. For patients with advanced UCEC tumor stages, limited treatments can be applied [11]. As a result, it is important to understand the progression of UCEC and to identify novel therapeutic targets in order to improve the survival rate of patients with endometrial cancer.

Bioinformatics is a rapidly growing discipline that can be applied to large datasets and can uncover the biological significance of cancer-related data [5, 12, 13]. Analysis of high-throughput RNA-sequencing data of UCEC revealed that the molecular motor Myosin Vb (MYO5B) was elevated at the level of mRNA and protein in the beginning stages of UCEC. MYO5B is a motor that treadmills on F-actin to deliver cargo to and from the apical membrane of cells. Although MYO5B has not previously been examined in UCEC, we speculated that its role in trafficking of nutrient transporters in the setting of normal homeostasis may be hijacked to promote cancer cells [14–17]. Cancer cells have an increased nutrient requirement to continue proliferating and these cells commonly uptake macromolecules from the environment via actin-dependent endocytic events [18]. Under normal conditions, MYO5B participates in epithelial actin-dependent events and recycling so it is possible that MYO5B plays a significant role in nutrient uptake in UCEC. We predicted that elevated MYO5B in UCEC would influence cancer metabolism reprogramming and result in a worse prognosis.

## Materials and methods

### Uterine cancer statistical sources

The incidence and mortality of uterine cancer were collected from the Global Burden of Disease (GBD) 2019 study via the Global Health Data Exchange (GHDx) website (http://ghdx.healthdata.org/gbd‐results‐tool) as previously described [19]. The Global Health Data Exchange (GHDx) database harbors data from multiple cancer databases and contains 21 regions nested within seven super‐regions, covering 204 countries or territories within the 21 regions. Data was collected from the explore option after the input of “B.1.16 Uterine Cancer”.

### mRNA analysis

Several databases were used to query mRNA in large publicly available datasets. MYO5B mRNA in pan-cancer and UCEC was examined using the UALCAN Database (http://ualcan.path.uab.edu) as previously described [20]. The database contains demographics on patients age, ethnicity, tumor type (endometroid, serous, mixed, *etc*.), tumor stage, tumor grade, tumor histology, menopause status and survival. Data was collected using the input gene MYO5B in UCEC TCGA dataset. Genotype-Tissue Expression (GTEx) Portal v8 (https://gtexportal.org/) was also used to examine the distribution of MYO5B mRNA in human tissue. The input for analysis was the gene MYO5B (gencode ID: ENSG00000167306.19) with the dbGaP accession number phs000424.vN.pN on 03/10/22. The GTEx Project is supported by the Common Fund of the Office of the Director of the National Institutes of Health, and by NCI, NHGRI, NHLBI, NIDA, NIMH, and NINDS. TNMplot (https://tnmplot.com) was used to confirm the distribution of MYO5B mRNA in UCEC [21]. Data was collected using the gene expression comparison function with the input MYO5B and comparing RNA-Seq data from tumor and normal tissue.

Mutation frequency of MYO5B in endometrial cancer was confirmed in COSMIC v95 (Catalogue of Somatic Mutations in Cancer https://cancer.sanger.ac.uk/) [22] The input for analysis was the gene MYO5B (gencode ID: ENSG00000167306.19) and mutations were identified using the COSMIC annotations for MYO5B in a genomic context. Mutations and mRNA correlation were examined in the TIMER2.0 database (https://cistrome.shinyapps.io/timer/) [23]. Data was collected from the mutation function using the input UCEC and MYO5B. A mutation map of MYO5B was generated using the cBioPortal v4.1.1 portal (https://www.cbioportal.org/) as previously described [24, 25]. Briefly, the UCEC TCGA datasets Firehose Legacy, Nature 2013 and PanCancer Atlas were selected and queried by the gene MYO5B. The output was alteration frequency by mutation type and using the mutation function, the lollipop mutation map was generated.

Mutation type, frequency, and copy number variation (CNV) of UCEC tumors from the Cancer Genome Atlas (TCGA) (https://www.cancer.gov/about-nci/organization/ccg/research/structural-genomics/tcga/studied-cancers) was observed in the MutationMapper module. MEXPRESS (https://mexpress.be/) was also used to examine MYO5B mutations and methylation [26, 27]. The input for the analysis of MYO5B and UCEC. The program generates a mutation map based on mutation type, frequency and copy number and correlates the data to expression. Correlations between genes were examined using the TIMER2.0 database (https://cistrome.shinyapps.io/timer/) [23, 28, 29] using the Gene_Corr feature with the input of two genes of comparison. The data was analyzed with a Spearman’s correlation. Correlations between genes was confirmed using the GEPIA2 Database (http://gepia2.cancer-pku.cn/).

### Methylation analysis

MYO5B promoter methylation in UCEC was examined in the MET500 dataset using the UALCAN Database (http://ualcan.path.uab.edu) [20]. Data was collected using the input gene MYO5B in UCEC TCGA dataset. Methylation was examined in the context of patients age, ethnicity, tumor type (endometroid, serous, mixed, *etc*.), tumor stage, tumor grade, and tumor histology. Beta values indicate the DNA methylation level. Values range from 0 (unmethylated) to 1 (fully methylated). A Beta-value: 0.7–0.5 is considered hypermethylated and a Beta-value: 0.3–0.25 is considered hypomethylated.

### Protein and survival analysis

Protein levels of MYO5B were obtained using the UALCAN Database (http://ualcan.path.uab.edu) which draws from the Clinical Proteomics Consortium for Cancer Analysis (CPTAC) dataset [20]. Data was collected using the input gene MYO5B in UCEC CPTAC dataset. Protein analysis was obtained based on information on patients age, ethnicity, tumor type (endometroid, serous, mixed, *etc*.), tumor stage, tumor grade, tumor histology, and survival. The z-value represents the standard deviation of the median sample for different cancer types. Survival curves were generated using the cBioPortal v4.1.1 portal (https://www.cbioportal.org/) [24, 25] and the UALCAN Database (http://ualcan.path.uab.edu).

### Pathway and metabolism analysis

The STRING website (https://string-db.org/) was queried using the term “MYO5B” and organism “homo sapiens” as previously described [30]. STRING pathways were downloaded and examined in Cytoscape. Protein interactions were examined using the following parameters: Full STRING Network with evidence-based network edges set a medium confidence (0.4) and no more than 10 interactors in the 1^st^ and 2^nd^ shell. The lines between interactions are depicted based on confidence of the interaction with the thickest lines indicating the highest confidence and the thinnest lines indicating the lowest confidence in the interaction. Pathway analysis was performed with the top 20 positively correlated genes associated with MYO5B in UCEC (**Table 1**), as identified in the UALCAN database, using the Gene Set Cancer Analysis (GSCALite) program (http://bioinfo.life.hust.edu.cn/web/GSCALite/) [31]. The global percentage and relationship network were generated using the gene set. The Cancer Cell Metabolism Gene DB (http://bioinfo.uth.edu/ccmGDB/) [32] was used to visualize metabolic pathways in UCEC using the Cancer type search. The DAVID Bioinformation Database 2021 (https://david.ncifcrf.gov) was used to annotate the functions of the top 50 genes found to positively correlate with MYO5B in UCEC according to the UALCAN database (**Table 2**).

**Table 1:** Top 50 genes positively correlated with MYO5B in UCEC tumors.

Genes	PearsonCC
MPZL3	0.56
PLEKHA7	0.56
CELSR1	0.56
VPS4B	0.54
DSG2	0.54
KLF5	0.52
SMAD2	0.52
CTNND1	0.51
KIAA1632	0.51
RREB1	0.5
MARVELD2	0.5
LNX2	0.5
ATP8B1	0.5
CAPN1	0.49
SOCS6	0.49
OCLN	0.49
MBD1	0.49
VEZF1	0.49
C18orf25	0.48
AGAP1	0.48
HIPK1	0.48
WDR7	0.48
KIAA1671	0.48
ELF1	0.48
CTNNA1	0.48
SMAD4	0.48
PTPRK	0.48
USP38	0.47
RNF111	0.47
SHROOM3	0.47
VANGL1	0.47
PPL	0.47
NARS	0.47
MAML2	0.47
PSEN1	0.47
ANXA2P2	0.47
KIAA1468	0.47
SH3D19	0.47
CDC42BPB	0.47
RSPRY1	0.47
ARHGEF12	0.47
RNF6	0.47
KLF3	0.46
MYO5C	0.46
ATXN1L	0.46
TNFAIP1	0.46
SGPP2	0.46
ASXL2	0.46
SEC24B	0.46

**Table 2:** Functional annotation of top genes positively associated with MYO5B in UCEC.

Cluster 1 (Enrichment Score: 4.3)
cell junction
cell-cell junction
zonula adherens
Leukocyte transendothelial migration
Cluster 2 (Enrichment Score: 2.27)
cell junction
cell-cell adhesion
beta-catenin binding
cadherin binding
cell adhesion
Cell adhesion
cell surface
Cluster 3 (Enrichment Score: 1.79)
Zinc-finger
Zinc
metal ion binding
Metal-binding
Cluster 4 (Enrichment Score: 1.68)
CROSSLNK:Glycyl lysine isopeptide (Lys-Gly)
Isopeptide bond
Ubl conjugation
Cluster 5 (Enrichment Score: 1.51)
protein ubiquitination
ubiquitin-protein transferase activity
Zinc finger, RING-type
RING
DOMAIN:RING-type
ZN_FING:RING-type
Zinc finger, RING/FYVE/PHD-type
Cluster 6 (Enrichment Score: 1.39)
DOMAIN:PH
Pleckstrin homology domain
PH
Pleckstrin homology-like domain
Cluster 7 (Enrichment Score: 1.38)
cell junction
Cell junction
apical plasma membrane
Cell membrane
plasma membrane
Membrane
TOPO_DOM:Cytoplasmic
TOPO_DOM:Extracellular
integral component of membrane
TRANSMEM:Helical
Disease variant
Transmembrane helix
Transmembrane
Cluster 8 (Enrichment Score: 1.23)
nucleoplasm
positive regulation of transcription
transcriptional activator activity
positive regulation of transcription from RNA polymerase II promoter
Transcription regulation
Transcription
negative regulation of transcription from RNA polymerase II promoter
Activator
transcription factor activity, sequence-specific DNA binding
nucleus
DNA-binding
DNA binding
transcription factor complex
Ubl conjugation
Nucleus
ZN_FING:C2H2-type 1
RNA polymerase II core promoter proximal region sequence-specific DNA binding
ZN_FING:C2H2-type 2
chromatin
RNA polymerase II transcription factor activity, sequence-specific DNA binding
ZN_FING:C2H2-type 3
regulation of transcription from RNA polymerase II promoter
Zinc finger C2H2-type/integrase DNA-binding domain
DOMAIN:C2H2-type
sequence-specific double-stranded DNA binding
cell differentiation
ZnF_C2H2
Cluster 9 (Enrichment Score: 1.18)
transforming growth factor beta receptor signaling pathway
negative regulation of transcription, DNA-templated
negative regulation of cell proliferation
Cluster 10 (Enrichment Score: 1.13)
protein deubiquitination
DNA binding
regulation of transcription, DNA-templated
chromatin binding
Cluster 11 (Enrichment Score: 0.9)
Gastric cancer
Hippo signaling pathway
Pathways in cancer
identical protein binding
Cluster 12 (Enrichment Score: 0.68)
negative regulation of gene expression
macromolecular complex
positive regulation of gene expression
Cluster 13 (Enrichment Score: 0.36)
cell adhesion
Concanavalin A-like lectin/glucanase, subgroup
TOPO_DOM:Cytoplasmic
TOPO_DOM:Extracellular
Signal
CARBOHYD:N-linked (GlcNAc. . .) asparagine
Disulfide bond
Glycoprotein
Cluster 14 (Enrichment Score: 0.24)
NP_BIND:ATP
ATP binding
ATP-binding
Nucleotide-binding

### Immunofluorescence staining, imaging and mean fluorescence intensity analysis

A tissue microarray of endometrium cancer tissue and adjacent tissue was purchased from US Biomax (Cat# UT243a; US Biomax). The tissue microarray contained cases of endometrioid adenocarcinoma with matched adjacent endometrium tissue. The biopsy core sections were 5 μm thick and were from formalin fixed paraffin embedded blocks. Adjacent tissue present on the tissue microarray was collected from 1.5 cm away from the tumor. The tissue microarray was deparaffinized in histoclear (Cat# HS-200; National Diagnostics) and rehydrated in a series of ethanol solutions followed by water. Antigen retrieval was performed using a pressure cooker and citrate buffer. Slides were heated in the pressure cooker for 30 minutes and allowed to cool on ice at room temperature. Serum-free protein block (Cat# X0909; Dako) was used to block the slides for 1.5 hours at room temperature. Myosin 5b antibody (Cat# NBP1-87746; Novus Biologicals) was diluted 1:100 in antibody diluent with background reducing components (Cat#S3022; Dako). The primary antibody was added to the tissue microarray and incubated overnight at 4°C in a humidified chamber. The following day the tissue microarray was washed in 3 changes of PBS and the secondary antibody conjugated to a Cy3 fluorophore (donkey-anti-rabbit Cy3; #711-165-152; Jackson Immunoresearch) was added to the tissue at 1:200 dilution in antibody diluent (Cat#S0809; Dako) for 1 hour at room temperature in the dark. Hoechst was diluted 1:1000 in PBS to stain the nuclei for 5 minutes following the secondary antibody incubation. The tissue microarray was washed 3 times in PBS and coverslipped using ProLong Gold Antifade (Cat#P36934; Thermo Fisher Scientific) and allowed to dry prior to imaging.

Images were acquired on a Zeiss Axio Imager microscope equipped with an Axiovision digital imaging system using a 20x objective. The exposure was held constant for all acquired images. Single channel grayscale TIFF images were exported using the Axiovision digital imaging system. Image J software was used to calculate the mean fluorescence intensity of matched adjacent and tumor tissue. Adobe Photoshop was used to merge grayscale images and to assemble figures.

## Results and discussion

**Fig 1:**
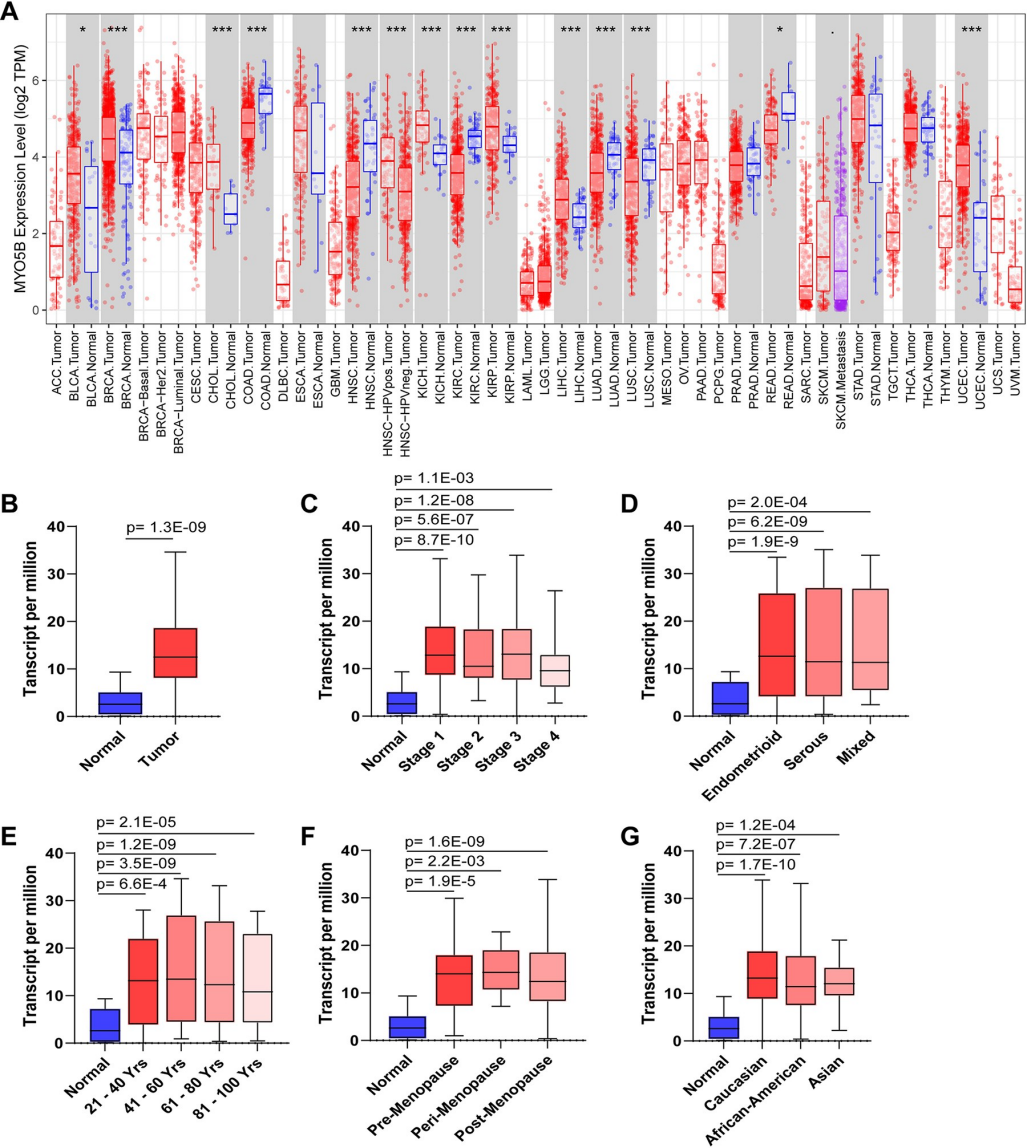
MYO5B mRNA is elevated in uterine corpus endometrial carcinoma (UCEC) patients. **A.** The expression of MYO5B in various control tissues and tumor types. *p<0.05, **p<0.001, ***p<0.0001. mRNA expression in transcript per million of MYO5B in normal and **B.** grouped UCEC tumor samples; **C.** stage 1, 2, 3 and 4 UCEC tumors; **D.** endometroid, serous, or mixed tumor types; **E.** patient age; **F.** menopause status, or patient ethnicity.

Myosin Vb (MYO5B) is a protein involved in the transport of materials within cells. MYO5B traverses on F-actin to deliver components to and from the apical membrane and it plays vital roles in epithelial polarity, intracellular trafficking, and cellular recycling. RNA sequencing of pan-cancers identified that MYO5B expression varies depending on the cancer type (**Fig 1A**). Interestingly MYO5B has lower expression in Colon Adenocarcinoma (COAD), Head-Neck Squamous Cell Carcinoma (HNSC), Kidney Renal Clear Cell Carcinoma (KIRC), Lung Adenocarcinoma (LUAD), Lung Squamous cell Carcinoma (LUSC), and Rectum Adenocarcinoma (READ), but was elevated in urothelial bladder carcinoma (BLCA), Cholangiocarcinoma (CHOL), Kidney Chromophobe (KICH), KIRP (kidney renal papillary cell carcinoma), and Uterine Corpus Endometrial Carcinoma (UCEC) compared to controls. Examination of the OMICS data in the Cancer Genome Atlas (TCGA) revealed a significantly elevated level of MYO5B expression in normal tissue compared to UCEC tumors (**Fig 1B**). This elevated MYO5B expression was observed in all stages of cancer (Stage 1–4 according to the International Federation of Gynecology and Obstetrics (FIGO) staging system; **Fig 1C**). Most endometrial cancers are classified as endometrioid, followed by the serous and mixed types [2]. We observed elevated MYO5B transcript in all tumor types (**Fig 1D**), suggesting that MYO5B is not restricted to stage or type. Although women are commonly diagnosed with UCEC at >60 years of age, we found high MYO5B expression at all ages, including the 21–40 and 41–60 years old (**Fig 1E**). Likewise, we found no difference in menopauses status (**Fig 1F**). MYO5B elevation was independent of ethnicity as it was elevated in Caucasians, African Americans, and Asians (**Fig 1G**). We interpret this data to indicate that MYO5B is a central component of UCEC tumors and participates throughout the tumor life span.

Increased MYO5B expression in UCEC was positively correlated with several genes which could be clustered by functional annotation (**Tables 1 and 2**). These genes grouped into 14 clusters, and included clusters based on cell junctions, cell adhesion, transcriptional regulators, DNA-binding, ATP-binding and pathways in cancer.

**Fig 2:**
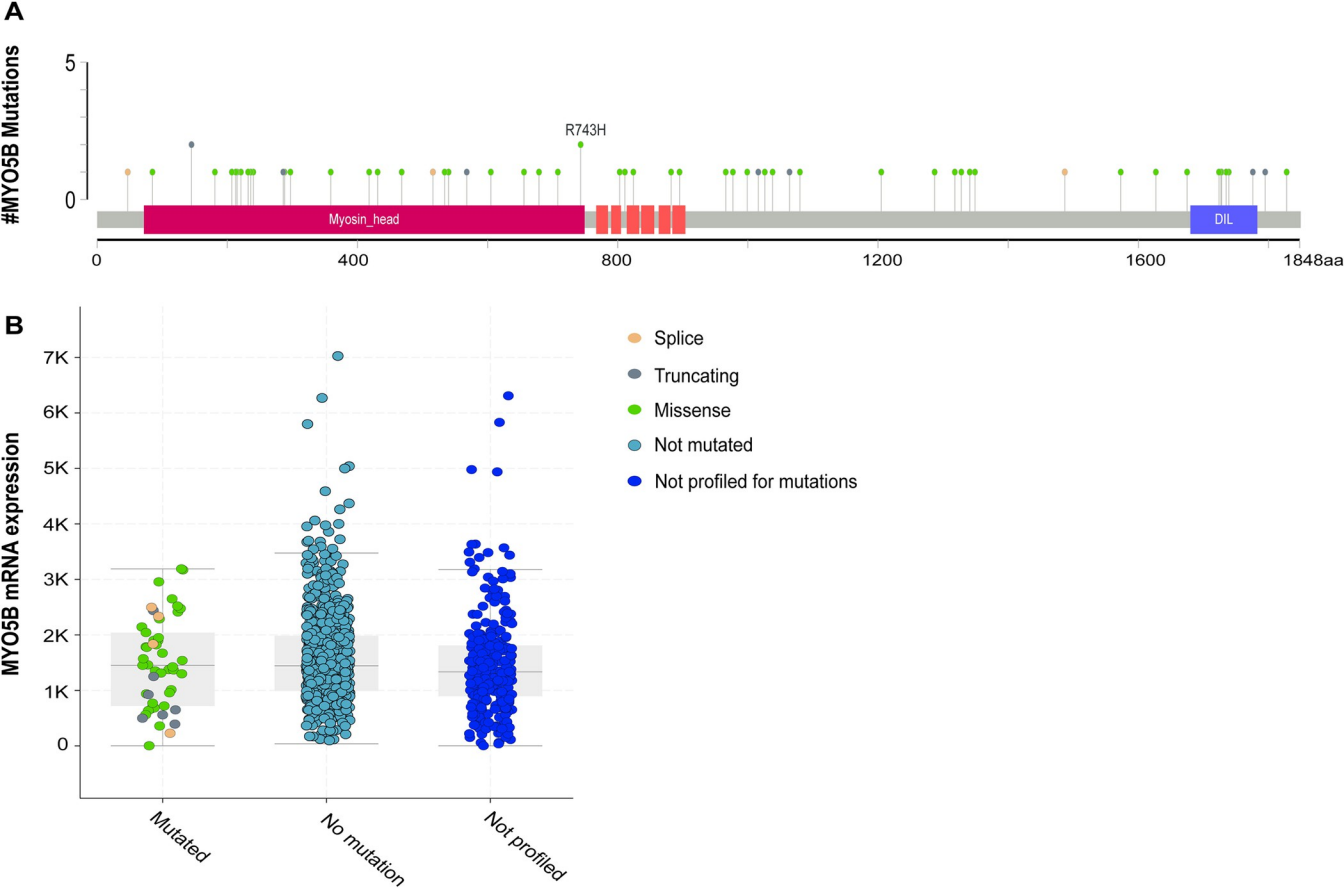
MYO5B is not commonly mutated in UCEC patients. **A.** Graphic of the MYO5B gene with lollipops identifying mutation sites **B.** MYO5B mRNA expression in the context of mutations.

Previous groups have identified mutations in MYO5B in pheochromocytoma and paraganglioma (PPGL) that increase its expression and enhance cancer progression [33, 34]. As a result, we sought to determine if similar activating mutations were present in MYO5B in UCEC samples. A mutation map revealed that mutants were widely distributed along MYO5, with several occurring in the Myosin head region (**Fig 2A**). However, none of the previously identified mutations that were associated with increased MYO5B were identified in UCEC. Moreover, the mutated MYO5B in UCEC did not result in significantly higher mRNA expression (**Fig 2B**), suggesting mutations in MYO5B were not responsible for the elevated levels of MYO5B observed in UCEC samples.

**Fig 3:**
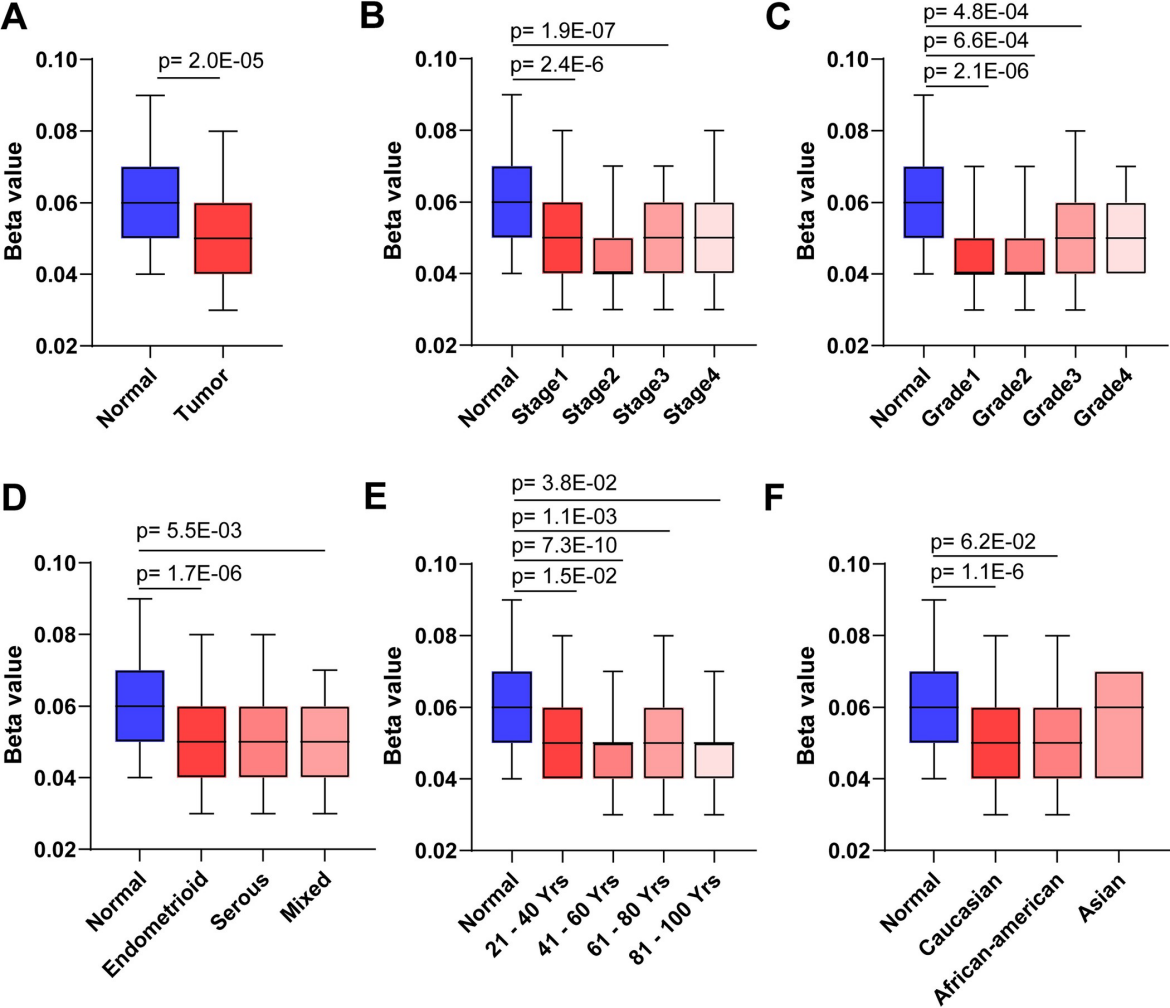
The MYO5B promoter is under-methylated in UCEC patients. The methylation status of the MYO5B promoter as denoted by beta-score in the following groups: **A.** normal and grouped UCEC tumor samples; **B.** stage 1, 2, 3 and 4 UCEC tumors; **C.** tumor grade, **D.** endometroid, serous, or mixed tumor types; **E.** patient age; **F.** menopause status, or patient ethnicity.

In gastric cancer and acute lymphoblastic leukemia, MYO5B is epigenetically silenced by aberrant DNA methylation [35–37]. Importantly, removal of methylation in gastric cancer cells with 5-aza-2’-deoxycytidine and trichostatin A enhances MYO5B expression [36]; suggesting that methylation status could be associated with elevated MYO5B in UCEC. Methylation analysis revealed that UCEC tumors were under-methylated compared to normal controls (**Fig 3A**). Similar to MYO5B mRNA data, we found that MYO5B was undermethylated in multiple tumor stages (**Fig 3B**), tumor grades (**Fig 3C**), and tumor types (**Fig 3D**). MYO5B under-methylation was also independent of age (**Fig 3E**) and ethnicity (**Fig 3F**); suggesting that lower methylation status of MYO5B drives MYO5B expression in UCEC.

To the best of our knowledge, we are the first to report that lower methylation status of MYO5B in UCEC correlates with increased MYO5B expression. Interestingly, Letellier *et al*. reported that colorectal cancer (CRC) cell lines and primary human CRC tumor samples did not show any tumor specific methylation of MYO5B or any differences in MYO5B methylation between healthy and cancerous tissue [37]. The authors found that in a CRC patient cohort there was a methylation-independent loss of MYO5B expression that aligned with CRC disease expression. These data suggest that MYO5B expression and methylation is likely tumor specific.

**Fig 4:**
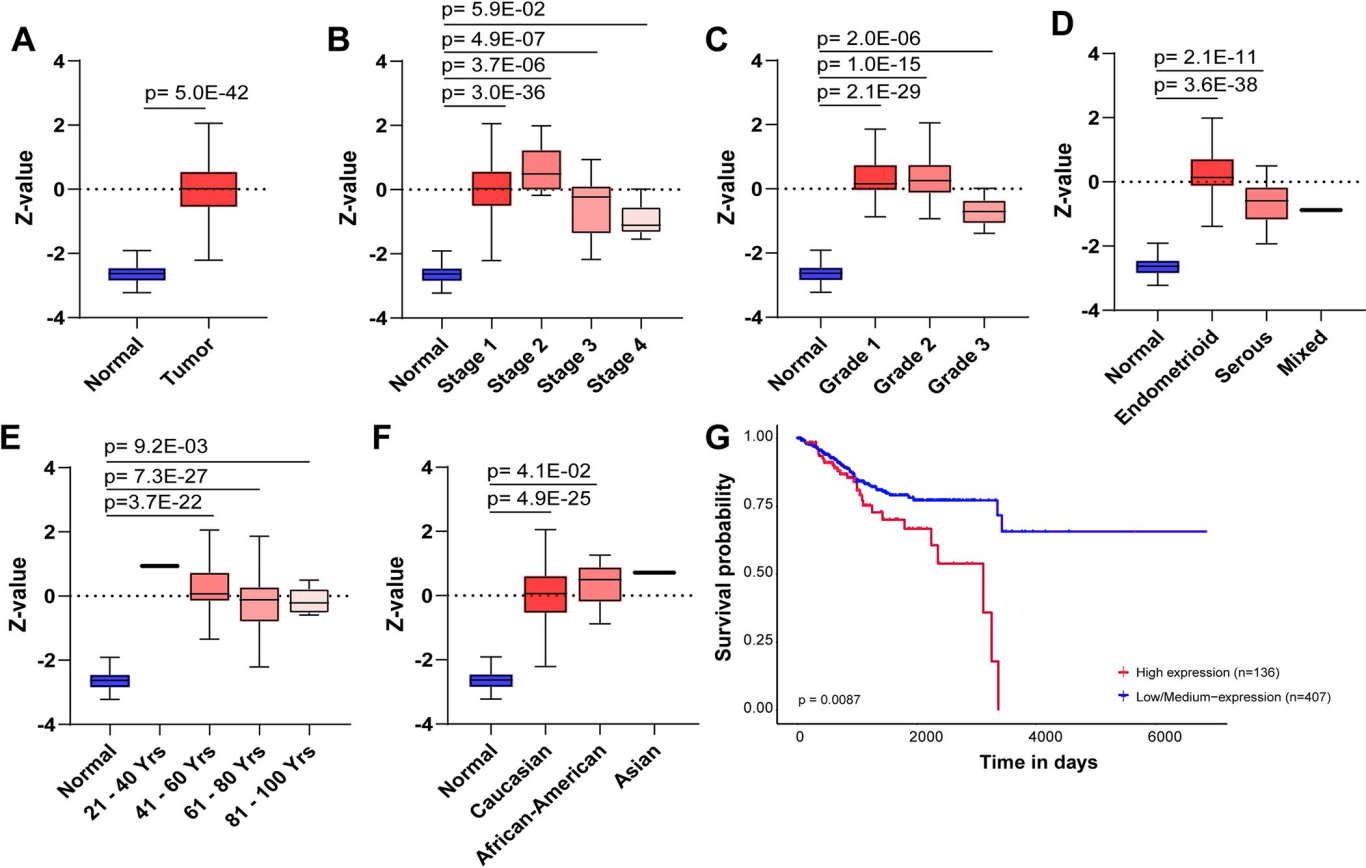
MYO5B protein levels are elevated in UCEC patients. Protein levels of MYO5B as determined by mass spectrometry-based proteomics (z-value) in normal and **A.** grouped UCEC tumor samples; **B.** stage 1, 2, 3 and 4 UCEC tumors; **C.** tumor grade; **D.** endometroid, serous, or mixed tumor types; **E.** patient age; **F.** menopause status, or **G.** patient ethnicity.

To confirm that MYO5B mRNA expression correlated with protein status, we examined MYO5B protein levels in UCEC samples using the Clinical Proteomics Consortium for Cancer Analysis (CPTAC) dataset. Mirroring our gene expression data, we found that MYO5B protein was elevated in UCEC tumors compared to normal controls (**Fig 4A**). And consistent with our MYO5B mRNA data, we found that MYO5B protein was increased in all tumor stages (**Fig 4B**), tumor grades (**Fig 4C**), and tumor types (**Fig 4D**). MYO5B protein was also elevated in all age groups (**Fig 4E**) and ethnicities (**Fig 4F**). Increased MYO5B expression correlated with worse survival rates, with all patients with high MYO5B passing before 4,000 days (**Fig 4G**).

**Fig 5:**
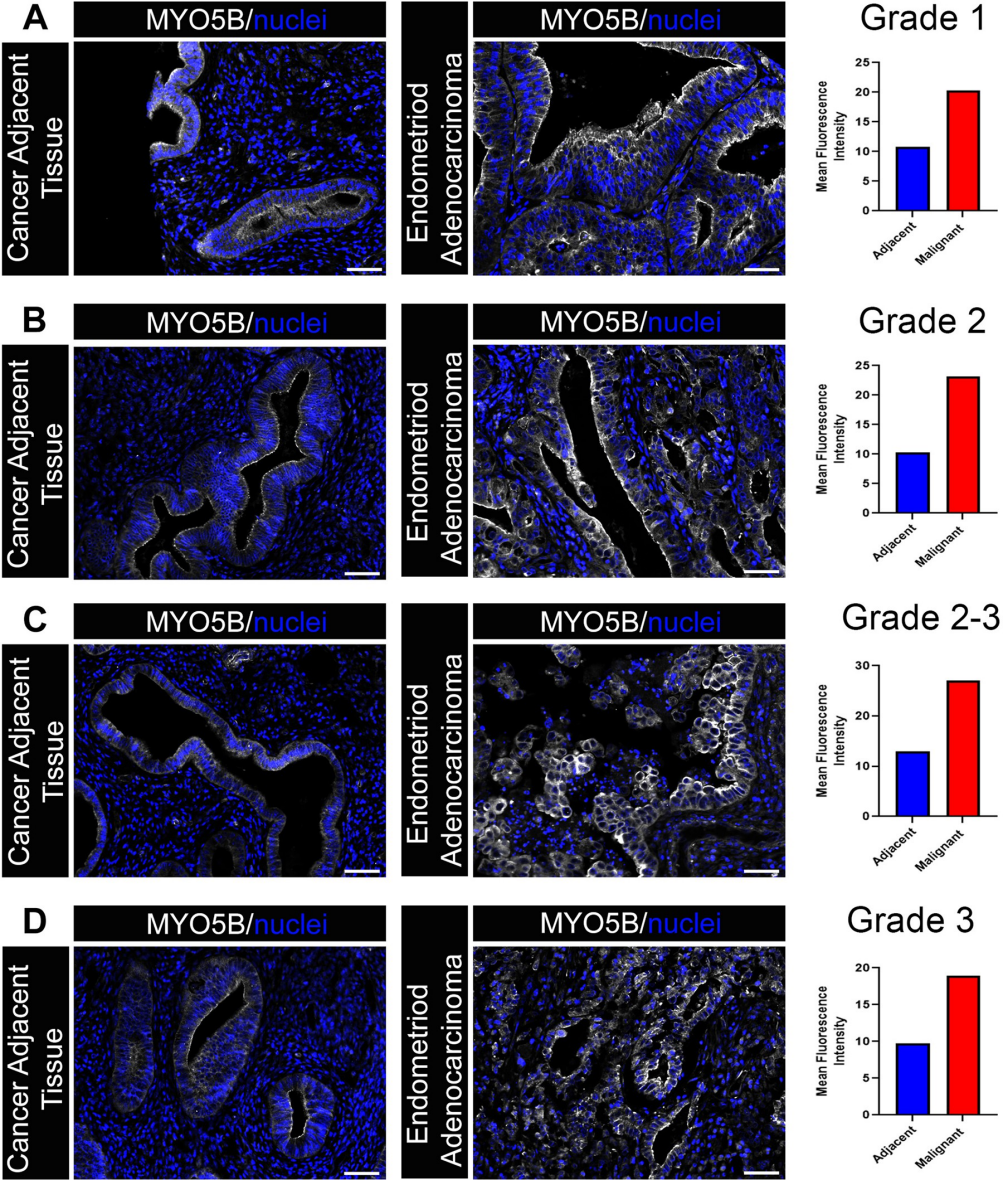
MYO5B protein is elevated in endometroid tumors, a subset of UCEC tumors. Immunostaining of endometroid tumors and adjacent uninvolved regions of endometrium. MYO5B is denoted in grey and nuclei in blue for **A.** Grade 1 tumors, **B.** Grade 2 tumors, **C.** Mixed Grade 2/3 tumors, and **D**. Grade 3 tumors.

To determine where MYO5B was localized in the endometrium, we immunostained endometroid tumors, one of the two subsets of UCEC tumors, and adjacent control tissue. Immunostained tissue revealed that MYO5B is present at low levels in the epithelium of the endometrium in control tissue (**Fig 5A–5D**). In contrast to adjacent controls, MYO5B was substantially elevated in the epithelium of endometroid tumor samples, with significant staining being observed on the apical membrane. Elevated levels of MYO5B were observed in Grade 1 (**Fig 5A**), Grade 2 (**Fig 5B**), Grade 2/3 (**Fig 5C**) and Grade 3 (**Fig 5D**) tumors. Apical staining of MYO5B is commonly observed in other cell types where MYO5B is involved in regulating actin dynamics and transporting cargo including nutrient channels. The localization of MYO5B in tumors at the apical membrane of the epithelium suggests that MYO5B may be participating in a similar role in nutrient transport in UCEC.

**Fig 6:**
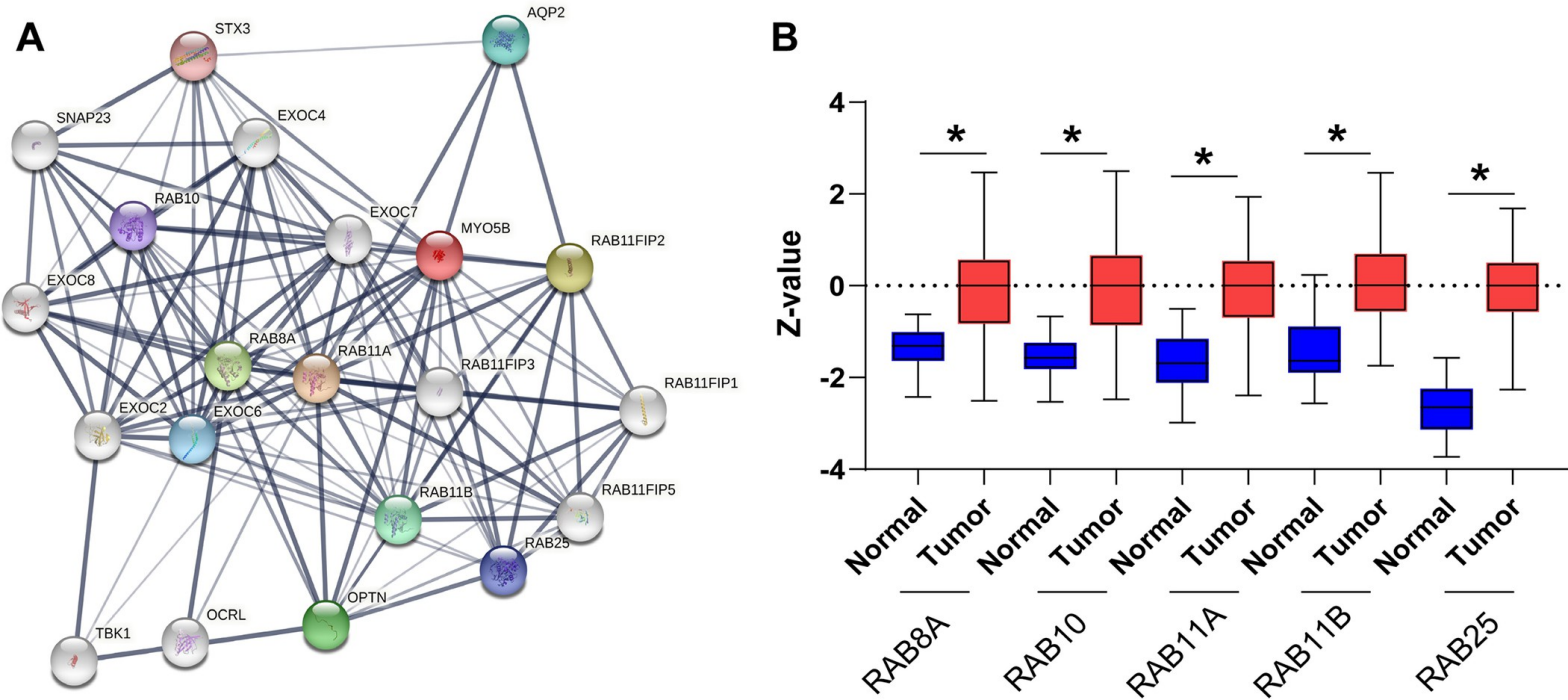
Protein networks indicate MYO5B closely interacts with RAB binding partners. **A.** Protein-protein interaction networks based on MYO5B (red circle). Active interactions are depicted by the thickness of the lines, with thicker lines indicating stronger confidence of the interaction. **B.** Protein levels of normal and grouped UCEC tumor samples for RAB8A, RAB10, RAB11A, RAB11B, and RAB25. *p<0.05.

Analysis of protein-protein interactions with MYO5B in normal tissue revealed that MYO5B interacts with multiple Rab GTPases (**Fig 6A**). Rab GTPases are highly conserved regulators of vesicular transport in cells [38]. Each Rab GTPase is localized to a different membrane compartment, where it regulates different trafficking routes and ensures cargo is delivered to the correct location [38, 39]. MYO5B is known to bind to certain Rab GTPases and traverse along actin to deliver the cargo. In the setting of cancer, Rab GTPases play a vital role in vesicle trafficking and are associated with invasion, migration, and metabolism [40]. The protein-protein network indicated that MYO5B had significant connections with Rab8A, Rab10, Rab11A, Rab11B and Rab25 proteins. Examination of the protein levels in normal and UCEC tumors revealed that UCEC tumors had elevated levels of all MYO5B associated Rab GTPases (**Fig 6B**). These data support the notion that MYO5B likely participates in vesicle transport in the setting of UCEC.

**Fig 7:**
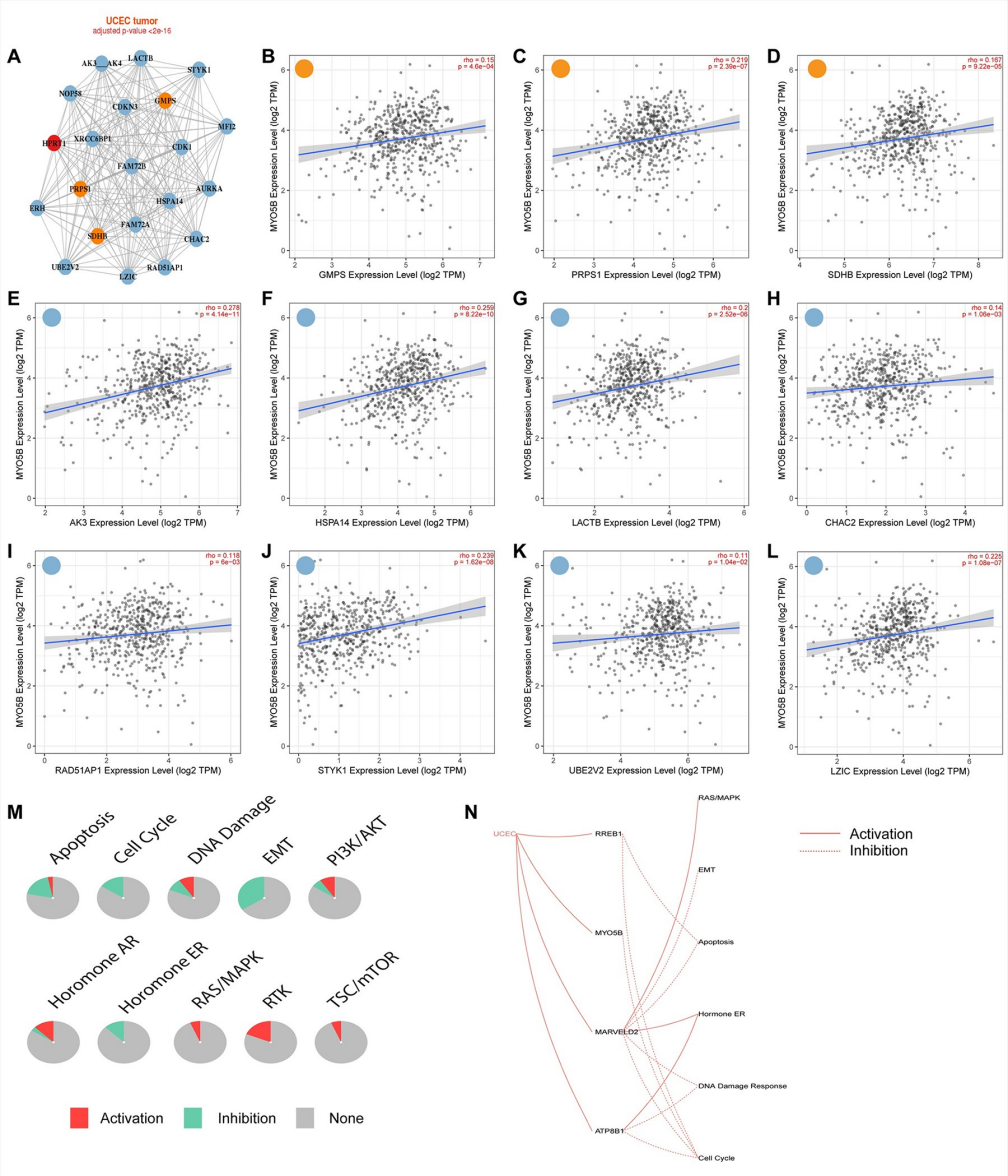
MYO5B mRNA positively correlated with several metabolism related genes and cell cycle in UCEC patients. A. Visualization of a metabolic pathway in UCEC patients with HPRT1 (red dot) as the inserted gene. Metabolism genes are visualized as orange dots and associated genes are visualized as blue dots. MYO5B was found to correlate with metabolism genes B. GMPS; C. PRPS1; and D. SDHB. Positive correlations with MYO5B were also observed with other genes including E. AK3; F. HSPA14; G. LACTB; H. RAD51AP1; J. STYK1; I. UBE2V2 and L. LZIC. M. Pathway Activity module of the top genes positively correlated with MYO5B mRNA expression in UCEC tumors. The pathway activity module presents the difference of genes expression between pathway activity groups (activation (red) and inhibition (green)) based on pathway scores. The pie graphs represent the global percentage of cancers in which a gene has an effect on the pathway among 32 cancer types. N. Relationship network based on the top genes positively correlated with MYO5B mRNA expression in UCEC tumors. The network indicates the relationship between genes and pathways by a line connection. Solid line indicates activation, dashed line indicates inhibition.

Rab-mediated vesicle trafficking is known to support uncontrolled cell growth in the setting of cancer [40]. Many cancers reprogram their metabolism to match the alternations in nutrient uptake [41]. We speculated that elevated vesicle trafficking by MYO5B and RABs may shift the metabolic pathways in UCEC since MYO5B is known to play a vital role in positioning of transporters required for nutrient acquisition in other epithelial cells [16, 17]. Visualization of metabolic pathways in UCEC with the input HPRT1 revealed several connections with metabolism related genes (orange circles: GMPS, PRPS1, and SDHB) and other genes (blue circles) (**Fig 7A**). Upregulation of MYO5B expression was found to positively correlate with the increased expression of metabolism genes Guanine Monophosphate Synthase (GMPS), phosphoribosyl pyrophosphate synthetase (PRPS1) and Succinate Dehydrogenase Complex Iron Sulfur Subunit B (SDHB) (**Fig 7B–7D**). MYO5B also correlated with the expression of many other genes identified in the metabolic pathway (**Fig 7E–7L**), suggesting that MYO5B status is closely linked with cancer metabolism. We speculate that elevated levels of MYO5B protein may enhance the shuttling of cargo to and from the apical membrane and increase the nutrient availability of UCEC tumors. Elevated levels of nutrients in tumor cells have been associated with metabolic reprogramming [42]. Our data indicates that MYO5B is positively correlated with multiple metabolism genes, which we believe reflects the changing metabolism profile of UCEC.

Previous groups have identified that UCEC tumors are enriched in genes related to proliferation, MAPK signaling pathways, regulation of cell cycle and PI3K-Akt signaling [43]. Since we saw significant correlations between MYO5B and UCEC metabolism, we created a pathway relation network with the top 20 genes that were positively correlated with MYO5B in UCEC to determine if MYO5B and any of the connected genes influence these proliferation-related pathways (**Fig 7M, 7N**). The relation network revealed that MYO5B status was associated with activation of P13K/AKT, RAS/MAPK, RTK and mTOR; pathways which promote proliferation. MYO5B was also associated with inhibition of apoptosis and cell cycle. Pathway Analysis indicated that UCEC resulted in the activation of MYO5B as well as RREB1, MARVEL02 and ATP8B1 and that MYO5B directly inhibited cell cycle, while the other activated genes regulated additional cell functions (**Fig 7N**). Collectively, these data indicate that MYO5B may work in concert with other genes to regulate proliferation and cell cycle in UCEC.

Recently several studies have identified genetic and molecular components that can influence UCEC prognosis [44, 45]. These factors include: PI3K/AKT/mTOR signaling (proliferation), WNT signaling (proliferation), autophagy, immune interactions and cell-cycle and division genes [45–49]. Our computation analysis reveals that MYO5B also participates in concert with several of the genes that are known to influence UCEC. We identified that MYO5B is connected to inhibition of cell cycle. A previous study found that loss of MYO5B was associated with altered spindle orientation and cell division orientation [50]; indicating that MYO5B is involved in cell cycle regulation under homeostatic conditions. We also found that MYO5B was linked with genes involved in proliferation, including the PI3K/AKT/mTOR signaling pathway. It is possible that by shifting metabolism, MYO5B contributes to proliferation and cancer promotion.

**Fig 8:**
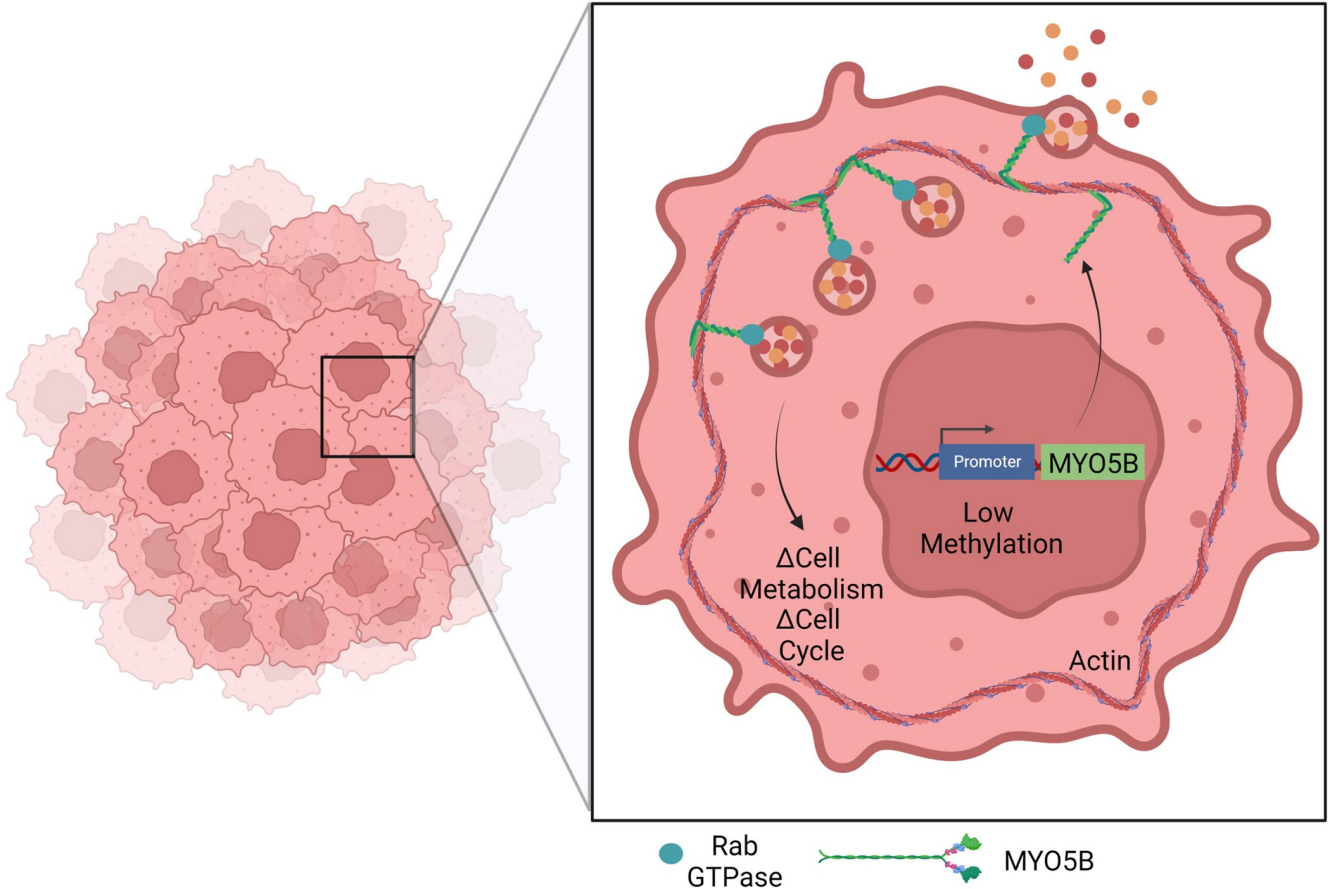
Model diagram of current findings. We speculate that low methylation of the MYO5B promoter enhances the gene expression of MYO5B and elevates levels of MYO5B protein. MYO5B binds to Rab GTPases to transport cargo in UCEC tumors and thereby contributes to altered cell metabolism and cell cycle. Created with BioRender.com.

High throughput sequencing technologies and public databases have provided a wealth of information for data mining. Using publicly available datasets, we report that non-diseased endometrium expresses low levels of MYO5B, while UCEC tumors express elevated levels of MYO5B transcript. Interestingly, the elevation in transcript was not linked to specific mutations, but rather to lower levels of methylation. Consistent with the mRNA data, we found elevated MYO5B protein in UCEC patients compared to healthy controls in a mass spectrometry-based proteomics database. Immunostaining of patient tumors confirmed increased levels of MYO5B at the apical membrane of UCEC tumors compared to adjacent non-diseased tissue and patients with elevated expression of MYO5B had worse survival. Alternations in MYO5B levels was linked to RAB GTPases and cell metabolism. We speculate that by shifting metabolism and cell cycle, MYO5B is connected to proliferation and cancer progression (**Fig 8**). These findings are among the first to demonstrate that MYO5B is elevated in UCEC and linked to poor prognosis.

The importance of these findings is highlighted by the fact that uterine cancer is the fourth most common cancer in females in the United States and the number of individuals diagnosed with uterine cancer is increasing each year [19]. Although UCEC is usually detected early, patients with advanced tumor stages at diagnosis have limited treatment options and poorer prognosis. Our work indicates that UCEC patients with elevated MYO5B have worse prognosis. These patients may benefit from inhibitors of MYO5B in combination with other drugs. Currently, it is not known whether MYO5B is elevated in uterine sarcoma, and we think exploration of this cancer would be a valuable comparison for UCEC.
